# Estrogen Degradation Metabolites: Some Effects on Heart Mitochondria

**DOI:** 10.3390/jox15050170

**Published:** 2025-10-18

**Authors:** Cristina Uribe-Alvarez, Elizabeth Lira-Silva, Lilia Morales-García, Natalia Chiquete-Felix, Francisco Javier Roldán-Gómez, Jesús Vargas-Barrón, José J. García-Trejo, Alejandro Silva-Palacios, Salvador Uribe-Carvajal, Natalia Pavón

**Affiliations:** 1 Department of Molecular Genetics, Institute of Cellular Physiology, National Autonomous University of Mexico, México City 04510, CP, Mexico; 2 Department of Pharmacology, National Institute of Cardiology Ignacio Chávez, Juan Badiano No. 1, Col. Sección XVI, Tlalpan, Mexico City 14080, CP, Mexico; 3 Outpatient Clinic, National Institute of Cardiology Ignacio Chávez, Juan Badiano No. 1, Col. Sección CVI, Tlalpan, Mexico City 14080, CP, Mexico; 4 Research Coordination, National Institute of Cardiology Ignacio Chávez, Juan Badiano No. 1, Col. Sección CVI, Tlalpan, Mexico City 14080, CP, Mexico; 5 Department of Biology, Faculty of Chemistry, National Autonomous University of Mexico, Mexico City 04510, CP, Mexico; 6 Department of Cardiovascular Biomedicine, National Institute of Cardiology Ignacio Chávez, Juan Badiano No. 1, Col. Sección XVI, Tlalpan, Mexico City 14080, CP, Mexico

**Keywords:** estrogen degradation metabolites, heart mitochondria, oophorectomy, OXPHOS uncoupling

## Abstract

Mitochondria play crucial roles in various cellular functions, including ATP production, apoptosis, and calcium homeostasis. Signaling pathways and hormones such as estrogens regulate the mitochondrial network through genetic, epigenetic, and metabolic processes. Estrogens increase the efficiency of mitochondrial oxidative phosphorylation by preventing uncoupling. Upon reaching menopause, when estrogen levels decrease, impaired mitochondrial function (uncoupled oxidative phosphorylation, lower ATP yields) is observed. Like all hormones in the body, estrogens undergo metabolic processing, resulting in estrogenic degradation metabolites (EDMs). These metabolites can form adducts with genomic and mitochondrial DNA and are of particular interest due to their potential role as carcinogens. Given that estradiol influences mitochondrial function, it is possible that EDMs may have an impact on heart mitochondria. To investigate this, we used isolated heart mitochondria from control and oophorectomized (mimicking menopausal stage) female Wistar rats of the same age. We found that mitochondria exposed to EDMs exhibited reduced coupling of oxidative phosphorylation and diminished ATP production, while increasing reactive oxygen species generation. Furthermore, these effects were significantly stronger in mitochondria from oophorectomized rats than in mitochondria from control (intact) rats. In addition, mitochondrial oxidative phosphorylation complex activities were differentially affected: complex I and ATPase activities decreased, while complex IV remained unaffected. We propose that exposure to EDMs promotes mitochondrial dysfunction in rats and that these effects are exacerbated by oophorectomy, a procedure commonly used to model the effects of menopause in women.

## 1. Introduction

Menopause is a relatively late event in the hormonal life of women that is characterized by anovulatory cycles and low estrogen levels. Menopause is frequently accompanied by hot flushes, mood changes, headaches, and sleep alterations, among other symptoms. It has been shown that estradiol (E2) decreases to ≤100 pmol/L as compared to 432–1893 pmol/L in pre-menopause. Pre-menopausal animal models and clinical studies show that estrogens exert strong cardiovascular protection. However, after menopause, when estrogens decline severely, cardiovascular morbi-mortality increases dramatically, making cardiovascular diseases the leading cause of death in menopausal women [1,2]. The molecular basis for this phenomenon is not well defined, although estrogen depletion has been postulated as a major cause [3,4].

In post-menopausal women, estrone-sulfate (E1S) is the predominant form of circulating plasma estrogen that metabolizes into its biologically active form, estrone (E1). In peripheral tissues, such as blood vessels, where aromatase activity is expressed, the primary precursors of estrogen synthesis are androstenedione and testosterone [5]. Up to a few years ago, estrogens were believed to be degraded into biologically inactive metabolites that lacked significant physiological effects. However, this idea has changed in recent years, as new data suggest that estrogen degradation metabolites (EDMs) may be biologically active [6]. Estrogen metabolism may include several biochemical modifications, such as sulfation, peroxidation, reduction, O-methylation, hydroxylation, and glucuronidation. While these processes are observed mainly in the liver, extrahepatic tissues such as the uterus, breast, kidney, brain, and pituitary can also degrade estrogen [7]. Estrogens can be metabolized into catechol estrogens through aromatic hydroxylation. Among these estrogen degradation products are 2-hydroxyestrogen (2-OH-E1) and 4 –hydroxyestrogen (4-OH-E1) [8]. Unlike estrone and estradiol, EDMs can bind covalently to DNA, leading to oxidative DNA damage [9,10,11,12]. In addition, nucleophilic sites in proteins also suffer EDM damage [13]. Studies demonstrate that mitoplasts (i.e., mitochondria without outer membrane) transform stilbene estrogen and 2-hydroxyestradiol to reactive metabolites that form DNA adducts in breast tumors [9]. In some cases, estrone and estradiol could act as epigenetic carcinogens that stimulate abnormal cell proliferation via estrogen receptors [14].

It is important to notice that cancer is not the leading cause of death in menopausal women; instead, cardiovascular diseases are. In previous research, we showed that there are at least 8 EDMs whose concentration was modified in menopausal women at risk of having a stroke, or with a previous stroke, when compared with those from healthy women, and with those found in women with breast cancer [15]. Additionally, women with breast cancer or at risk of (with positive markers) have higher levels of EDMs when compared to healthy women [16]. Estrogens are known to cause alterations in Ca^2+^ homeostasis, affect oxidative phosphorylation (OXPHOS), morphology, phospholipid content, fission-fusion, calcium homeostasis, apoptosis, and antioxidant production [17,18,19,20,21,22,23,24]. However, the effect of EDMs on heart mitochondria has not been reported. Thus, in this work, we compare the effect of EDMs in mitochondria from female intact rats (Ctrl, modeling pre-menopause) to those from oophorectomized (Oopho, modeling post-menopause) rats. We found that Oopho mitochondria exhibited lower respiratory controls (RC) and ΔΨ. These results, together with others described in this work, suggest that mitochondria from Oopho hearts undergo primary damage due to the absence of estrogens throughout several months. In this scenario, EDMs are probably more detrimental to the mitochondria of Oopho rats (modeling post-menopausal women) than to intact control mitochondria, raising the question of whether these mitochondrial alterations may resemble the conditions observed during menopause in women.

## 2. Materials and Methods

### 2.1. Animals

Twenty female 4-week-old Wistar rats were randomly assigned to Ctrl group (10) or Oopho group (10). They were fed with standard lab chow and water ad libitum, and kept under 12 h light-dark cycles. After three months, the ovaries of the rats in the Oopho group were removed surgically. This eliminated estrogens and simulated a post-menopause state. Previous studies have shown that this technique decreases estrogens significantly [25]. The Ctrl group went through the same manipulation without removal of the ovaries. Six months after surgery, both groups of rats of the same age were euthanized with sodium pentobarbital 100 mg/kg i.p. Experiments were conducted in agreement with ethical rules and guidelines from the Instituto Nacional de Cardiología, México (Record No. 14-865).

### 2.2. Reagents

All reagents were analytical grade. Estriol (E3) and 17β-estradiol (E2) were from Sigma (St. Louis, MO, USA). EDMs: 2-hydroxyestrone (2OHE1) was from Sta Cruz Biotechnology (Dallas, TX, USA), estrone-3-methyl-ether (3MOE1) was from Sigma (Darmstat, Germany); 4-methoxy-β-estradiol (4MEOE2) was from Chem-Cruz (Huissen, The Netherlands); 17-β-estradiol-3-methyl-ether (3MEOE2) was from Sigma-Aldrich Chemistry (Darmstat, Germany). Reagent stocks were diluted in 1 mg/mL concentration in ethanol according to their solubility. Based on their presence in post-menopausal women, an estrogen mixture (ALL) was prepared by mixing 3 equal parts of E3, E2, and EDMs as indicated.

### 2.3. Isolation of Heart Mitochondria

Rats from both groups (Ctrl and Oopho) were euthanized with 100 mg/kg i.p. sodium pentobarbital. Hearts were collected individually and cut into small pieces, and washed in cold isolation buffer (125 mM KCl, 10 mM EDTA, 10 mM Tris, pH 7.3) until the washing solution became clear. Afterwards, they were incubated for 10 min with proteinase K, 2 mg/g (Sigma, Darmstat, Germany, P6556). Digested samples were centrifuged at 10,000 rpm, and the remaining pellet was homogenized in a Potter–Evelheim homogenizer and centrifuged at 2000 rpm, to pellet the debris. The supernatant was transferred to a clean tube, and mitochondria were separated by differential centrifugation as indicated elsewhere [26]. Mitochondria were divided into two; one fraction was used immediately to measure oxygen consumption, transmembrane potential, and ROS production, while the remaining was frozen at −70 °C. All procedures were carried out at 4 °C. Protein was determined by the Bradford method [27].

### 2.4. EDM Incubation

Mitochondria, 10 mg protein, were incubated in a 1.5 mL Eppendorf tube with 1 ng of each one of the 7 EDMs per mg of mitochondrial protein, in a final volume of 100 µL of isolation buffer for 10 min. An additional sample was incubated with only saline (no EDMs) as a control and was marked as N/T (non-treated). For each heart, both in Ctrl and Oopho groups, we incubated 3 different samples for each EDM. Incubation of mitochondria with EDMs was timed with the oxygraph: when we were halfway through an oximetry trace, the next sample was prepared to ensure that all samples had the same incubation time.

### 2.5. Oxygen Consumption and Membrane Potential Measurements

Oxygen consumption and the mitochondrial transmembrane potential (ΔΨ) were measured simultaneously in an OROBOROS high-resolution oxygraph (Innsbruck, Austria) equipped with a Clark electrode and an O2k-Fluo LED2-Module (excitation LED 465 nm) at 25 °C. The 2 mL volume chamber was adjusted to 1.5 mL using the OROBOROS stoppers(Inssbruck, Austria). After the incubation described in Section 2.4, mitochondria (0.5 mg protein/mL) were added to a 1.5 mL chamber containing 1.5 mL of respiration buffer (125 mM KCl, 3 mM phosphate, 2 mM MgCl_2_, 10 mM HEPES, pH 7.3) plus 2 μM safranine-O. State 4 was initiated with 10 mM pyruvate-malate-glutamate, and then 2.5 mM ADP was added to induce State 3 [26,28]. Respiratory controls (RC) were calculated as the quotient between the rate of oxygen consumption in state 3 (phosphorylating state) over the rate in state 4 (resting state). At the end of each trace, 4 μM CCCP was added to uncouple respiration. A Supplementary Materials was added, showing the traces that were obtained in each group (Supplementary Figure S1). After every measurement, the Oroboros chambers were washed twice with ethanol and twice with respiration media to ensure they were clean.

### 2.6. Complex IV Activity

To evaluate complex IV activity, we measured oxygen consumption using 5 mM ascorbate, 10 μM Tetramethyl-phenylene-diamine (TMPD), and 0.1 μM Antimycin [29]. After the oxygen consumption trace stabilized, 1 mM cyanide (KCN) was added to evaluate nonspecific oxygen consumption.

### 2.7. High-Resolution Clear-Native Polyacrylamide Gel Electrophoresis (hrCN-PAGE) and In-Gel Enzymatic Activities

Mitochondria were incubated for 10 min with 1 ng/mg protein of EDMs and stored at −70 °C. hrCN-PAGE was performed as described in [30]. Briefly, treated mitochondria were re-suspended in sample buffer (750 mM aminocaproic acid, 25 mM imidazole, pH 7.0) and solubilized with 2 mg *n*-dodecyl-β-D-maltoside (laurylmaltoside, LM)/mg protein at 4 °C for 30 min, and centrifuged at 17,500 rpm for 1 h 15 min at 4 °C. 50 μg of protein from the supernatants were loaded into 4–12% (*w/v*) polyacrylamide gradient gels. After electrophoresis, Coomassie blue G-250 stain, in-gel NADH dehydrogenase (NADH-DH), and ATPase activities were performed as in [30,31]. Densitometry was performed using Image J (1.49v) software (NIH, USA).

### 2.8. Complex I (NADH-Ubiquinone Oxidoreductase) Activity

Complex I activity was measured by following the absorbance decrease in NADH at 340 nm (ε = 6.22 mmol^−1^ cm^−1^) in a POLAR star Omega microplate reader. Reaction mixture contained 100 µM NADH, 3 mg/mL BSA (bovine serum albumin), 300 µM KCN, 0.05% Triton X-100, and 0.5 mg/mL of isolated mitochondria in 0.2 mL of 50 mM phosphate buffer pH 7.5. The mixture was incubated for 5 min, and 60 µM ubiquinone-1 (Sigma, cat. no. C7956) in DMSO was added. The reaction was monitored for 10 min [32]. 5 µM Rotenone was added to inhibit complex I, and remaining inhibitor-insensitive activities were subtracted from the data. The length of the microplate well was considered when calculating activity.

### 2.9. Complex V (ATP Synthesis) Activity

ATP synthase activity was measured by following the absorbance increase in NADPH at 340 (ε = 6.22 mmol^−1^ cm^−1^) in a POLAR star Omega. Reaction mixture was 250 mM sucrose, 50 mM glucose, 1 mM ADP, 5 mM MgCl_2_, 20 mM Pi, 0.05% Triton X-100, 0.5 mM NADP^+^, 10 mM pyruvate-glutamate-malate, and mitochondria 0.5 mg prot/mL. The mixture was incubated for 5 min. Then we added 160 µg/mL hexokinase plus 2 U/mL glucose-6-phosphate dehydrogenase [33]. To inhibit ATP synthesis, 5 µg oligomycin/mL was added, and the inhibitor-insensitive activity was subtracted. The activity was calculated based on the length of the microplate well.

### 2.10. Reactive Oxygen Species (ROS)

ROS production was measured in a Synergy HT multi-mode microplate reader, Biotek (Winooski, VT, USA), using Amplex Red [34]. Briefly, hydrogen peroxide formation was determined following resorufin fluorescence in 0.6 M mannitol, 5 mM MES (pH 6.8), 20 mM KCl, 10 mM phosphate, 1 mM MgCl_2_, 10 μM Amplex Red (Invitrogen, Molecular Probes, Eugene, OR, USA), 0.1 U/mL horseradish peroxidase and 100 U/mL superoxide dismutase, 0.5 mM NADH or 10 mM pyruvate/10 mM malate and 0.1 mg prot/mL of mitochondria in a final volume of 50 μL. Where indicated, EDMs were added at 1 ng/mg protein and incubated with mitochondria for 10 min before measurements. ROS production was measured for 5 min.

### 2.11. Statistical Analysis

Student’s *t*-test for unpaired data was used to compare the baseline variables of the groups. The ANOVA test was used to determine significant differences, which were then analyzed with the Newman-Keuls post-test to find intergroup differences. Data are expressed as the mean ± SD, and *p* < 0.05 was considered statistically significant. For each figure: * *p* < 0.01, ** *p* < 0.001 indicates statistically significant differences. Analysis was performed using the GraphPad Prism 6.0 statistical package.

## 3. Results

A comparative analysis of the effects of EDMs on cardiac mitochondria isolated from Ctrl versus Oopho rats was conducted. The physiological parameters evaluated were oxygen consumption, individual respiratory complex activities, mitochondrial membrane permeability, and ROS production.

**Figure 1:**
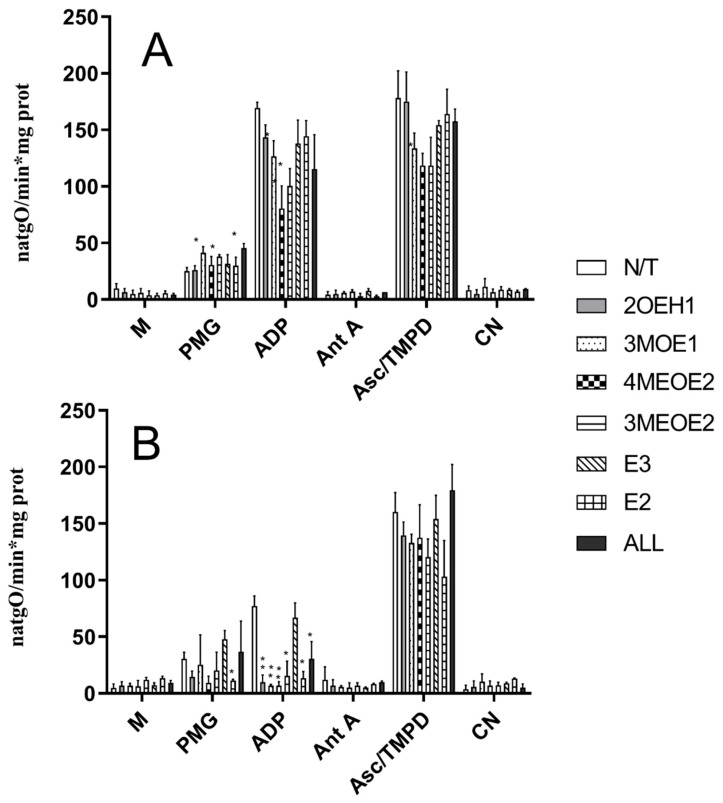
Effects of estrogens or EDMs on the rate of oxygen consumption in isolated heart mitochondria from Ctrl (**A**) and Oopho rats (**B**). Experimental conditions are as in Table 1. M indicates the addition of 0.5 mg prot/mL of mitochondria. The trace was started by adding 5 mM pyruvate-malate-glutamate (PMG). Where indicated, 2.5 mM ADP was added to induce state III. 1 µM Antimycin A (AntA) was used to inhibit Complex III activity. Ascorbate-TMPD was added to measure Complex IV activity. Then, 1 mM cyanide (CN) was added. N/T: no treatment, 2-hydroxyestrone (2OHE1), estrone-3-methyl-ether (3MOE1), 4-methoxy-β-estradiol (4MEOE2), 17-β-estradiol-3-methyl-ether (3MEOE2), Estriol (E3), 17β-estradiol (E2). Values expressed as natgO/min*mg prot, mean ± SD of *n* = 10 animals by group; * *p* < 0.01, ** *p* < 0.001 indicate significant differences.

The rate of oxygen consumption was measured using a mixture of NADH-producing substrates, pyruvate, malate, and glutamate (Figure 1, Table 1). Heart mitochondria isolated from Oopho rats had approximately a two-fold lower ADP-stimulated respiration than that of Ctrl rats (Figure 1), resulting in respiratory controls (RC) of 2.88 ± 0.58 versus 6.71 ± 0.99, respectively (Table 1).

Oxygen consumption was measured at 25 °C in respiration buffer (125 mM KCl, 3 mM phosphate, 2 mM MgCl_2_, 10 mM HEPES, pH 7.3). Mitochondria were added at 0.5 mg prot./mL, and 10 mM pyruvate-malate-glutamate was added to start the reaction. RC was calculated as the quotient between the rate of oxygen consumption in state 3 (2.5 mM ADP-stimulated respiration) and the rate of oxygen consumption in state 4 (resting state). We observed a decrease in O_2_ consumption during state III respiration, which reflects impaired ADP-stimulated oxidative phosphorylation and a reduction in ATP synthesis. In mitochondria from Ctrl rats exposed to the compounds 3MOE1, 4MEOE2, and 3MEOE2, the rate of oxygen consumption in state III respiration decreased, which led to a low respiratory control ratio (Figure 1A, ADP (* *p* < 0.01), Table 1). 2OHE1 and the non-degraded compounds E3 and E2 did not affect any OXPHOS coupling parameter (Figure 1). Comparatively, in Oopho rat heart mitochondria (Figure 1B), addition of E2, E3, or any of the EDMs led to a severe decline in state III respiration and lower respiratory control rates (Table 1). These results indicate that when heart mitochondria from Oopho rats were exposed to EDMs, mitochondrial homeostasis was severely disrupted, and their remaining OXPHOS coupling capacity was severely decreased (i.e., Respiratory control N/T 6.71 ± 0.99 vs. Oopho 2.88 ± 0.58). The activity of complex IV was slightly different between Ctrl and Oopho rats (Figure 1B, Asc-TMPD). Mitochondria from Ctrl rats were only affected by exposure to 4MEOE2, while in the Oopho group, mitochondria were affected exclusively by exposure to 3MEOE2. Interestingly, mitochondrial Complex IV activity was not affected by the mixture of EDMs (ALL) (see Section 2).

**Figure 2:**
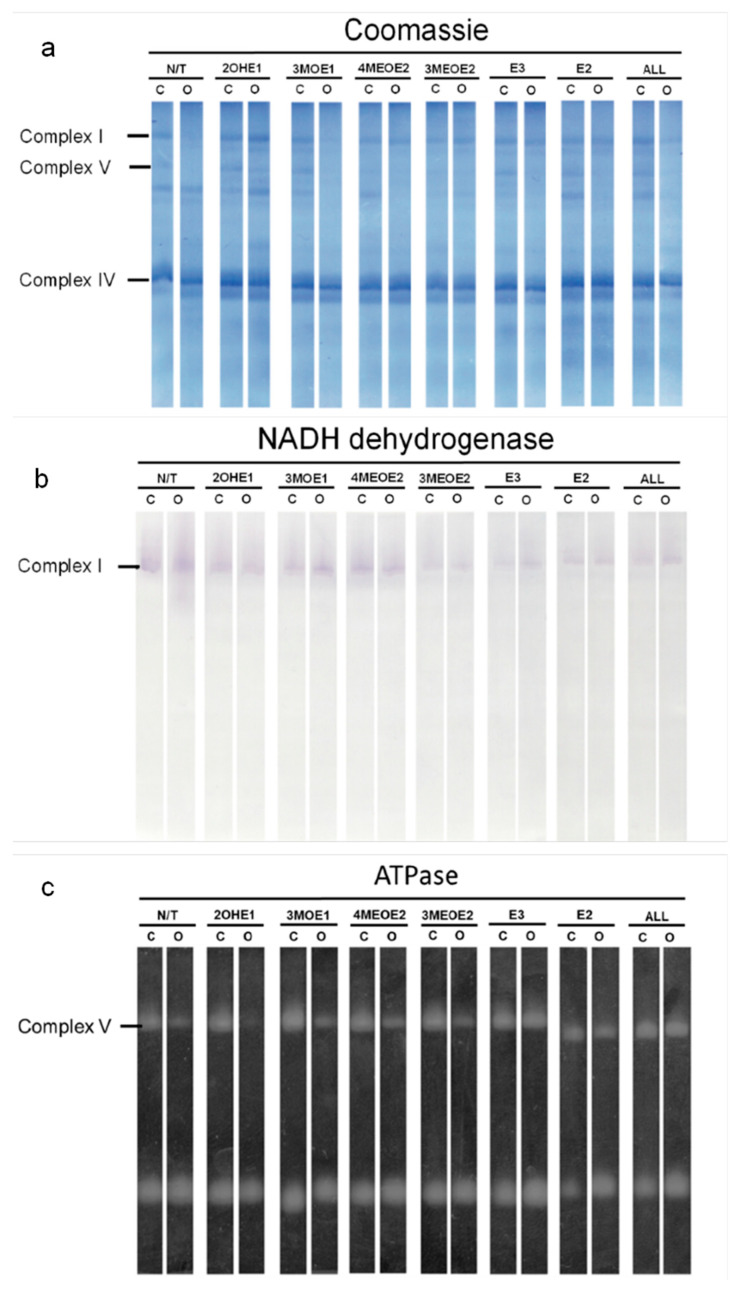
Concentration and activity of mitochondrial redox complexes in Ctrl and Oopho rat heart mitochondria. Representative images of (**a**) Coomassie-stained hrCN/BN-PAGE (**b**) Complex I: NADH dehydrogenase activity (NADH-DH). (**c**) ATPase activity (ATPase). N/T: no estrogen or EDMs added, 2-hydroxyestrone (2OHE1), estrone-3-methyl-ether (3MOE1), 4-methoxy-β-estradiol (4MEOE2), 17-β-estradiol-3-methyl-ether (3MEOE2), Estriol (E3), 17β-estradiol (E2).

The effects observed in the mitochondrial respiratory controls could be due to a deficiency in mitochondrial complex content, enzyme activity, or increased membrane permeability. Thus, we analyzed respiratory complex contents by BN-, hrCN-PAGE, and Coomassie staining (Figure 2a). In BN- and hrCN PAGE, respiratory complexes can be identified according to their molecular mass and their activity [30,35]. When comparing Ctrl versus Oopho mitochondria (Figure 2a, N/T), we observed that the concentration of Complex V (ATP synthase) and Complex I (NADH-DH) decreased in mitochondria from Oopho rats, while complex IV concentrations remained constant in Ctrl group (Figure 2a). Complex I concentration in Oopho mitochondria decreased in comparison with that observed in Ctrl group when both mitochondrial preparations were exposed to 3MOE1, 4MEOE2, and the mixture of EDMs (ALL) (Figure 2a, Coomassie). When measuring in-gel activity of Complex I, we found a decrease in its function when mitochondria from both Ctrl and Oopho rats were exposed to 3MOE1, 3MEOE2, E3, E2, and the mixture (ALL) (Figure 2b). Complex V also decreased in Oopho rat heart mitochondria and was further diminished when EDMs were added, except for E3, E2. This same pattern was observed in the in-gel activity measurements (Figure 2a,c). Since Complex IV was previously evaluated (Figure 1A,B, Asc-TMPD) with no significant change observed between Ctrl and Oopho groups, it was not analyzed further.

**Figure 3:**
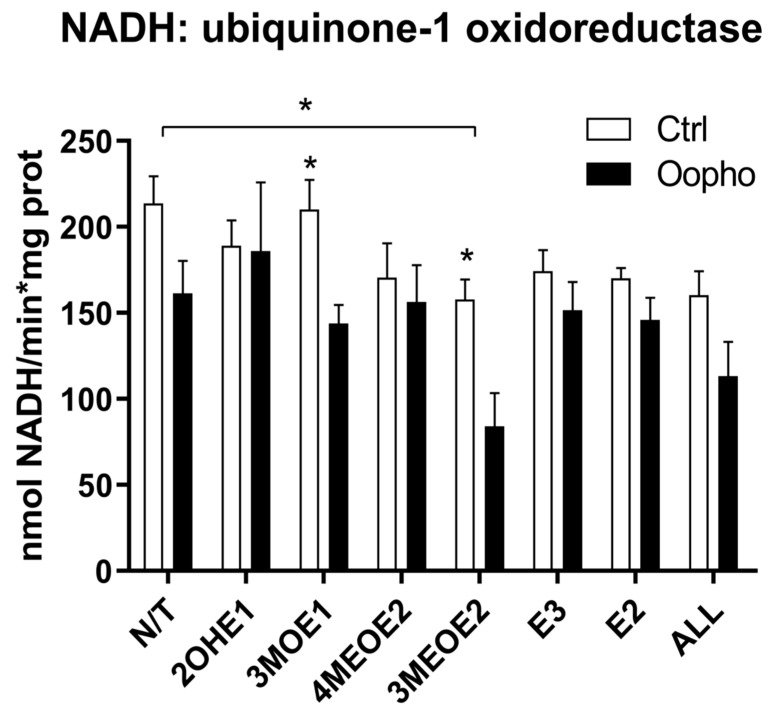
Complex I activity. Complex I (NADH-ubiquinone-1 oxidoreductase, NADH-DH) concentration and activity from Ctrl and Oopho rat heart mitochondria. Complex I activities were measured spectrophotometrically following the decrease in NADH at 340 nm (ε = 6.22 mmol^−1^ cm^−1^). White bars correspond to the Ctrl group and black bars to Oopho group. N/T: no estrogen or EDMs added, 2-hydroxyestrone (2OHE1), estrone-3-methyl-ether (3MOE1), 4-methoxy-β-estradiol (4MEOE2), 17-β-estradiol-3-methyl-ether (3MEOE2), Estriol (E3), 17β-estradiol (E2). Values expressed as mean ± SD of n = 10 animals by group; * *p* < 0.01, indicating significant differences.

**Figure 4:**
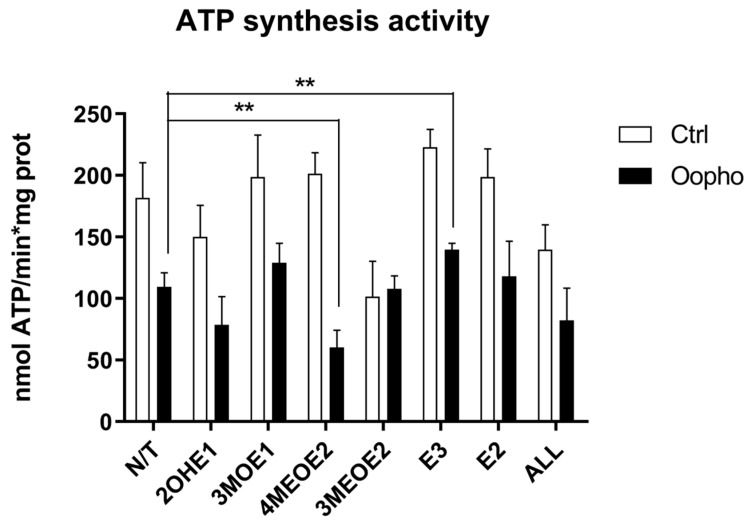
ATP synthase activity of Ctrl and Oopho rat heart mitochondria. ATP synthase activity was measured using an indirect method following NADPH production at 340 nm using an enzyme-coupled assay (ε = 6.22 mmol^−1^ cm^−1^). For every pair of columns, white bars represent Ctrl and black bars represent Oopho mitochondria. N/T: no estrogen, EDMs added: 2-hydroxyestrone (2OHE1), estrone-3-methyl-ether (3MOE1), 4-methoxy-β-estradiol (4MEOE2), 17-β-estradiol-3-methyl-ether (3MEOE2), Estriol (E3), 17β-estradiol (E2). Values expressed as mean ± SD of n = 10 animals by group; ** *p* < 0.001 indicates significant differences.

The in-gel activities of Complex-I and ATPase were both lower in Oopho rats, especially when mitochondria were exposed to EDMs; this was not observed for the Ctrl. To obtain quantitative values, Complex I and V were evaluated spectrophotometrically (Figure 3 and Figure 4). In basal conditions, Complex I activity was lower in the Oopho group than in the Ctrl group; the lowest NADH-DH activity for each group was observed when mitochondria were incubated with 3MEOE2 (Figure 3). Oopho mitochondria also exhibited lower NADH-DH activity than Ctrl when exposed to 3MOE1. In contrast, mitochondrial incubation with 4MEOE2, E3, or E2 did not modify the mitochondrial NADH-DH activity in either group.

Mitochondria depend on the transmembrane potential (ΔΨ) to synthesize ATP. Since ATP synthase activity was higher in Ctrl groups than in Oopho rats, we evaluated how mitochondrial exposure to EDMs modified ATP synthesis. Somehow unexpectedly, it was found that 2OHE1 and 3MEOE2 caused a decrease in ATP synthase activity in both groups (Figure 4). However, in Oopho mitochondria, the most detrimental effect was elicited by 4MEOE2.

**Figure 5:**
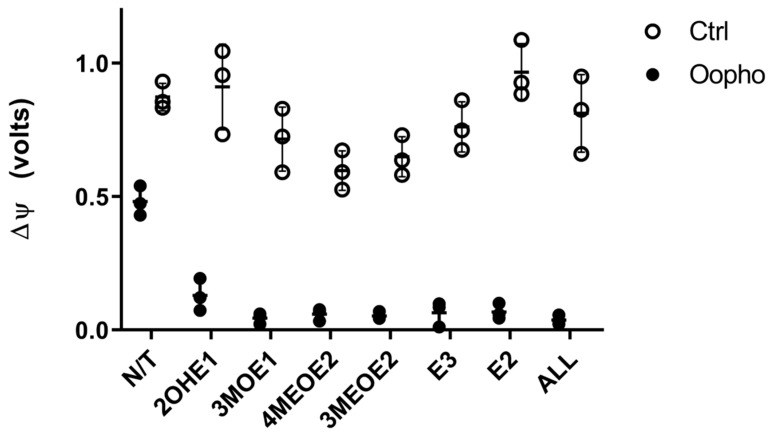
Effects of estrogens or EDMs on transmembrane potential (ΔΨ) of Ctrl and Oopho rat heart mitochondria. Transmembrane potential was measured in an Oroboros Respirometer, as fluorescence during the respiratory trace using a 465 nm excitation LED, after adding 5mM PMG and adding 6 μM of CCCP to eliminate the transmembrane potential. EDMs were added to a 1 ng/mg protein and incubated for 10 min before measuring. The buffer contained the fluorophore Safranine-O. ○ White circles correspond to Ctrl group and ● black circles to Oopho group. N/T: no estrogen or EDMs added, 2-hydroxyestrone (2OHE1), estrone-3-methyl-ether (3MOE1), 4-methoxy-β-estradiol (4MEOE2), 17-β-estradiol-3-methyl-ether (3MEOE2), Estriol (E3), 17β-estradiol (E2). Values expressed as a.u., mean ± SD of n = 3 animals by group. Student’s *t*-test for unpaired data was used for comparing groups, in all cases *p* < 0.005.

Since we observed a deficiency in ATP production, an effect on ΔΨ was plausible, so we decided to explore this possibility (Figure 5). Mitochondria of Ctrl rats established a two-fold higher ΔΨ than those from Oopho rats. Furthermore, when they were exposed to EDMs, in the Ctrl group, the ΔΨ decreased in response to 3MOE1, 4MEOE2, and 3MEOE2. However, mitochondria from Oopho rats exhibited a depletion of ΔΨ when any EDMs were added (Figure 5).

**Figure 6:**
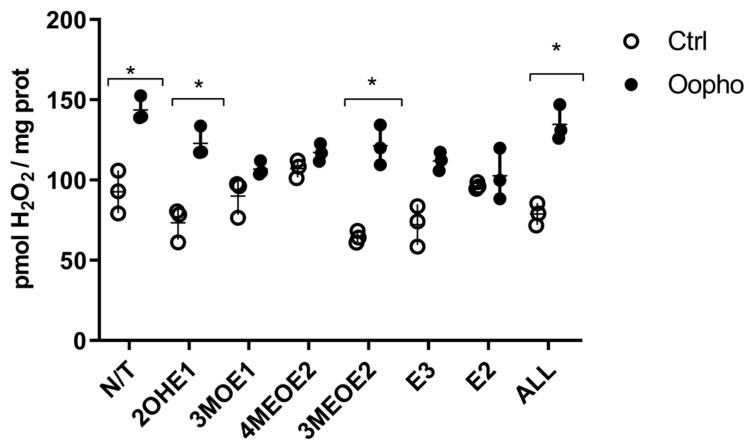
Effects of estrogen or EDMs on ROS production of Ctrl and Oopho rat heart mitochondria. ROS formation was followed using AmplexRed. ○ White circles correspond to Ctrl group and ● black circles to Oopho group. N/T: no estrogen or EDMs added, 2-hydroxyestrone (2OHE1), estrone-3-methyl-ether (3MOE1), 4-methoxy-β-estradiol (4MEOE2), 17-β-estradiol-3-methyl-ether (3MEOE2), Estriol (E3), 17β-estradiol (E2). Values expressed as mean ± SD of n = 3 animals by group. Student’s *t*-test for unpaired data was used for comparing columns, * *p* < 0.01 indicates significant differences.

The previous results suggested that the large decrease in ΔΨ observed could lead to overproduction of ROS. Therefore, we measured ROS under each condition (Figure 6). In mitochondria from Ctrl rats, ROS levels were lower than in mitochondria from Oopho rats. When EDMs were added, ROS production remained constant in the mitochondria from Ctrl rats. In contrast, in the presence of the EDMs 3MOE1, 4MEOE2, and E2 and E3, mitochondria from the Oopho group exhibited a similar level of ROS production than the Ctrl group, indicating that once the respiratory chain was uncoupled, the high production of ROS decreased to normal levels (Figure 6).

## 4. Discussion

The relationship between estrogens and mitochondrial function has been previously reported [25,36,37,38]. In the brain, estrogens regulate many of the key proteins involved in mitochondrial bioenergetics, including glucose transporters, succinate dehydrogenase (SDH), aconitase (Aco2), hexokinase (HK), pyruvate dehydrogenase (PDH) [39] and respiratory complexes I [40], III, and IV [41]. In the heart, estrogen deficit also modifies mitochondrial bioenergetics [25,37,42,43]. Estrogen receptors (ER) located in the plasma membrane migrate to nuclei and mitochondria, where they control protein expression [20,23]. After menopause, these tend to decrease, and thus cell responses to estrogens change [44,45]. In addition, EDMs probably interact differently with ER, thus explaining the observed response variations [46].

In our previous work, we showed that in heart mitochondria from Oopho rats, oxygen consumption decreased as compared to mitochondria from Ctrl rats [25]. This observation was corroborated here (Table 1, Figure 1A,B), suggesting that heart mitochondria isolated from Oopho rats are physiologically deficient as compared to those from Ctrl rats. In addition, we showed here that Ctrl mitochondria might be more resistant to EDM exposure. Oxygen consumption in state III refers to ADP-stimulated respiration and reflects the capacity of the respiratory chain to drive ATP synthesis. High oxygen consumption in state III indicates that the respiratory chain and the ATP synthase are functionally coupled. In Oopho mitochondria, values for state III respiration were decreased, which resulted in a lower RCR value and limited ATP synthesis. Exposure to EDMs decreased the RCR of the Ctrl mitochondria group, but interestingly, the lowest RCRs from the Ctrl group (exposed to 4MEOE2 and 3MEOE2) were similar to the RCR of the Oopho mitochondria with no EDMs exposure, suggesting that menopause alone is quite detrimental for mitochondrial integrity. Furthermore, the EDMs that caused more damage to mitochondrial respiration and yielded lower RCRs—in both Ctrl and Oopho mitochondria—were the methylated compounds. Estrone-3-methyl ether (3MEOE2) is also found to be increased dramatically in the serum of post-menopausal women who have cardiovascular disease [15], yet little information on its toxicity is documented, indicating a big gap in women’s health research. Furthermore, incubation of mitochondria with E2 caused no significant differences in the respiration rate, ATP synthesis, or TMP. There is conflicting evidence about whether estrogens exert strong cardiovascular protection or not. Moreira et al. reported that in isolated heart mitochondria, E2 had a negative effect by increasing depolarization and reducing Ca^2+^ load capacity [28]. Meanwhile Borrás et al. and Pavón et al. observed that in isolated mitochondria, long period incubations (1 h–1 h 45 min) with E2 had beneficial effects on mitochondria [17,25]. Unlike the previous authors, we only exposed mitochondria to E2 for ten minutes, which might not have allowed the possible beneficial effects of estradiol, suggesting these are time-dependent. These might also contribute to why Oopho rat mitochondria, which have a significant loss of E2 for long periods, have very low RCRs. Notably, we selected short incubation times because the EDMs showed high toxicity and led to a rapid decrease in mitochondrial function. Respiratory activity of Complex IV was decreased in the presence of 3MOE1, 4MEOE2, and 3MEOE2, and remained unchanged for every other EDM, E3, and E2. This indicates that the concentration of the other EDMs used is not enough to induce damage in Complex IV isolated activity. We did not observe any significant decrease in the mixture of EDMs (ALL), indicating these compounds may be interacting amongst them and inhibiting each other’s actions, or the E2 present in the mixture may be exerting its protective effects, decreasing EDMs damage in complex IV (Table 1, Figure 1B and Figure 2b). While all reagents were obtained from reputable suppliers, and EDMs purity was >97% verified by HPLC, the reagents used for buffer preparation were used without further purification. We acknowledge that trace impurities—such as calcium—cannot be entirely ruled out and may have introduced minor, random effects on mitochondrial function or respiratory chain activity. The conditions of the experiments were kept constant, and all Ctrl vs. Oopho experiments were carried out with the same buffers, so we do not expect these trace impurities to significantly impact our conclusions. EDMs stock concentrations were solubilized to 1 mg/mL in 20% ethanol and further diluted in saline to 1 ng/μL. Ctrl and oophorectomized mitochondria were not incubated with traces of Ethanol. Further experiments could include exposing Ctrl and Oopho mitochondria to different concentrations of the 3 methylated compounds with and without E2, and with different concentrations of ethanol to observe if toxicity remains or is abolished.

The effects on RCRs could be due to a deficiency in mitochondrial complex content, so we measured the concentrations of the respiratory chain components and measured their activities. In Oopho mitochondria, we detected lower concentrations and activities of Complex I and Complex V. This confirms suggestions from our previous work where this behavior was noticed for the first time [26]. Similar effects of EDMs were observed in Ctrl mitochondria: Isolated Complex I and Complex V activities were decreased only when they were exposed to 3MEOE2. Likewise, Oopho mitochondria Isolated Complex I activity was not that different from the Oopho control group and the NADH:ubiquinone oxidase activity was only decreased by the presence of 3MEOE2. In contrast, Complex V activity was dramatically decreased between the Ctrl and the Oopho groups, and when exposed to any of the compounds. Interestingly, mitochondria from Oopho rats were far more sensitive to estrogen and the EDMs than mitochondria from Ctrl rats. As far as we know, this is the first report of a toxic effect of EDMs on ATP synthase.

In addition to the OXPHOS protein-related effects, EDMs may affect the mitochondrial membrane, contributing to membrane fluidity and permeability modifications [25]. These changes would be reflected in the ΔΨ, which powers the ATP synthase, leading to a deficiency in ATP production. Therefore, EDMs effects on the ΔΨ were explored. Consistent with all our data, Oopho mitochondria had a lower ΔΨ than Ctrl mitochondria, which is further lost when exposed to estrogens or EDMs. The lower ΔΨ can be reflected as RC uncoupling, low ATP, and high ROS production [47]. 3MOE1, 4MEOE2, and 3MEOE2 lowered the TMP in Ctrl mitochondria. But interestingly, the addition of any of the EDMs collapsed the ΔΨ in Oopho mitochondria. Long exposure to low levels of estrogen can cause a membrane composition rearrangement, resulting in an enhancement in lipid peroxidation [25,48,49]. These changes may lead to increased EDM toxicity and/or an increase in ROS (Figure 6).

Our results indicate that 3MOE1, 4MEOE2, and 3MEOE2 have an acute toxicity in mitochondria isolated from the heart of Oophorectomized rats, when compared to non-Oophorectomized rats. In vivo studies have proposed that replacing the estrogen lost in menopause could prevent conditions such as osteoporosis, cardiovascular disease, and cognitive function [50,51,52]. But this may not be fully accurate: while some menopausal symptoms were alleviated and osteoporosis prevention was observed, other instances, such as stroke, coronary heart disease, pulmonary embolism, and breast cancer, increased their potential risk when estradiol was administered in hormonal replacement therapy (HRT) [53]. Therefore, estradiol is not completely beneficial in isolated mitochondria nor as HRT. We also know that the effects of EDMs on cardiac mitochondrial function increase after menopause, and cardiovascular illnesses increase steeply [54]. It would be worthwhile to study whether the lack of E2 allows an increased interaction between the EDMs and the mitochondria or their estrogen receptors. Recent publications show that estrogens and EDMs can act as cervix, breast, and lung carcinogens [55,56,57,58]. Increased EDMs have been found in serum and urine samples from women with breast cancer [16].

Estrogen level equilibrium is lost at the onset of menopause, probably leading to increased cardiovascular pathologies. Our results suggest that heart mitochondrial dysfunction may also decrease at the onset of menopause, diminishing cardiovascular function. Hormonal therapy is recommended to eliminate symptoms such as hot flashes, dry skin, headache, mood disturbances, and sleep alterations. However, it must be carefully administered and supervised, since it has been associated with some kinds of cancers. EDMs are associated with vascular dysfunction [46]. This has been reported in patients with mild and severe pre-eclampsia, where an abnormal metabolism is found, and where EDMs accumulate [59]. Hence, EDMs lead to dilatation of some systemic vascular beds [60,61] through NO synthesis. These data suggest that EDMs, beyond their carcinogenic activity, have important cardiovascular effects at the mitochondrial level. We hope more knowledge will be available in the coming years.

## Figures and Tables

**Table 1:** Respiratory control rate (State III/State IV) of Ctrl and Oopho rats exposed to estrogens or different EDMs.

	Control	Oopho
N/T	6.71 ± 0.99	2.88 ± 0.58
2-hydroxyestrone (2OHE1)	5.55 ± 1.05	0.67 ± 0.17 **
Estrone-3-methyl-ether (3MOE1)	3.20 ± 0.72 *	0.51 ± 0.36 **
4-Methoxy-β-estradiol (4MEOE2)	2.61 ± 0.025 **	0.71 ± 0.082 **
17-β-estradiol-3-methyl-ether (3MEOE2)	2.62 ± 0.30 **	0.76 ± 0.11 **
Estriol (E3)	4.49 ± 0.67 *	1.42 ± 0.16 *
17-β-estradiol (E2)	5.05 ± 1.11	1.17 ± 0.52 *
ALL	2.24 ± 0.69	1.01 ± 0.61 *

Values are the average of State III/State IV ± SD. n = 10, * *p* < 0.01, ** *p* < 0.001.

## Data Availability

The original contributions presented in this study are included in the article/Supplementary Materials. Further inquiries can be directed to the corresponding authors.

## Supplementary Materials

The following supporting information can be downloaded at: https://www.mdpi.com/article/10.3390/jox15050170/s1, Figure S1: Illustrative oximetry traces; Figure S2: Concentration and activity of mitochondrial redox complexes.
