# Supplementary material for: Estrogen Degradation Metabolites: Some Effects on Heart Mitochondria
Source: J Xenobiot. 2025 Oct 18;15(5):170. doi: 10.3390/jox15050170 (PMC12565399; doi:10.3390/jox15050170)
Supplement: Supplementary file 1 [file jox-15-00170-s001.zip › jox-3815459-supplementary.pdf]

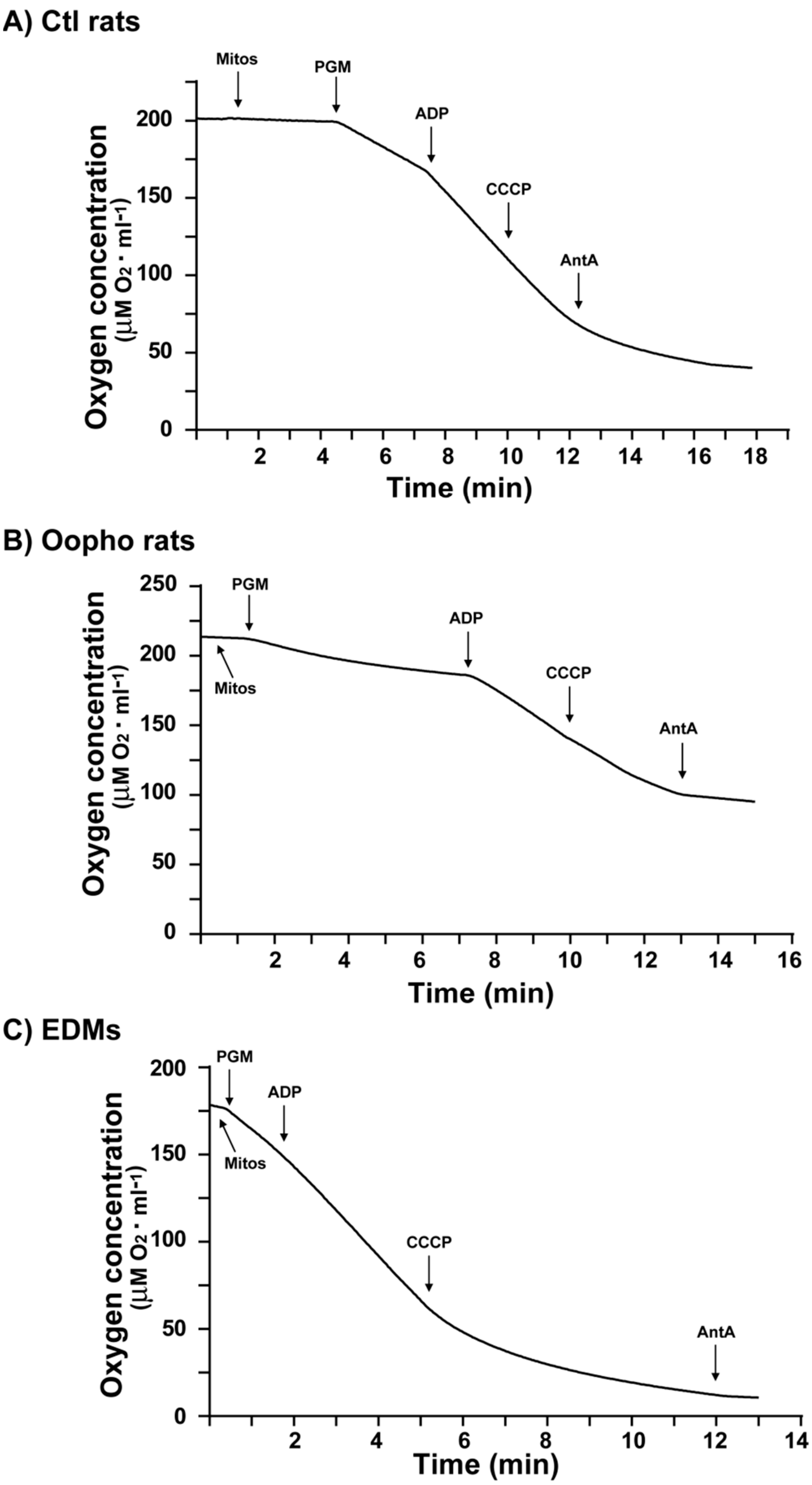

Figure S1. Illustrative oxymetry traces of mitochondria isolated from rats under different conditions, used to generate data in Table 1. Experimental conditions as in Table 1. A) Non-treated mitochondria from Controls; B) Non treated, mitochondria from Oophorectomized animals. C) Mitochondria from oophorectomized rats treated with a mixture of EDMs (ALL).

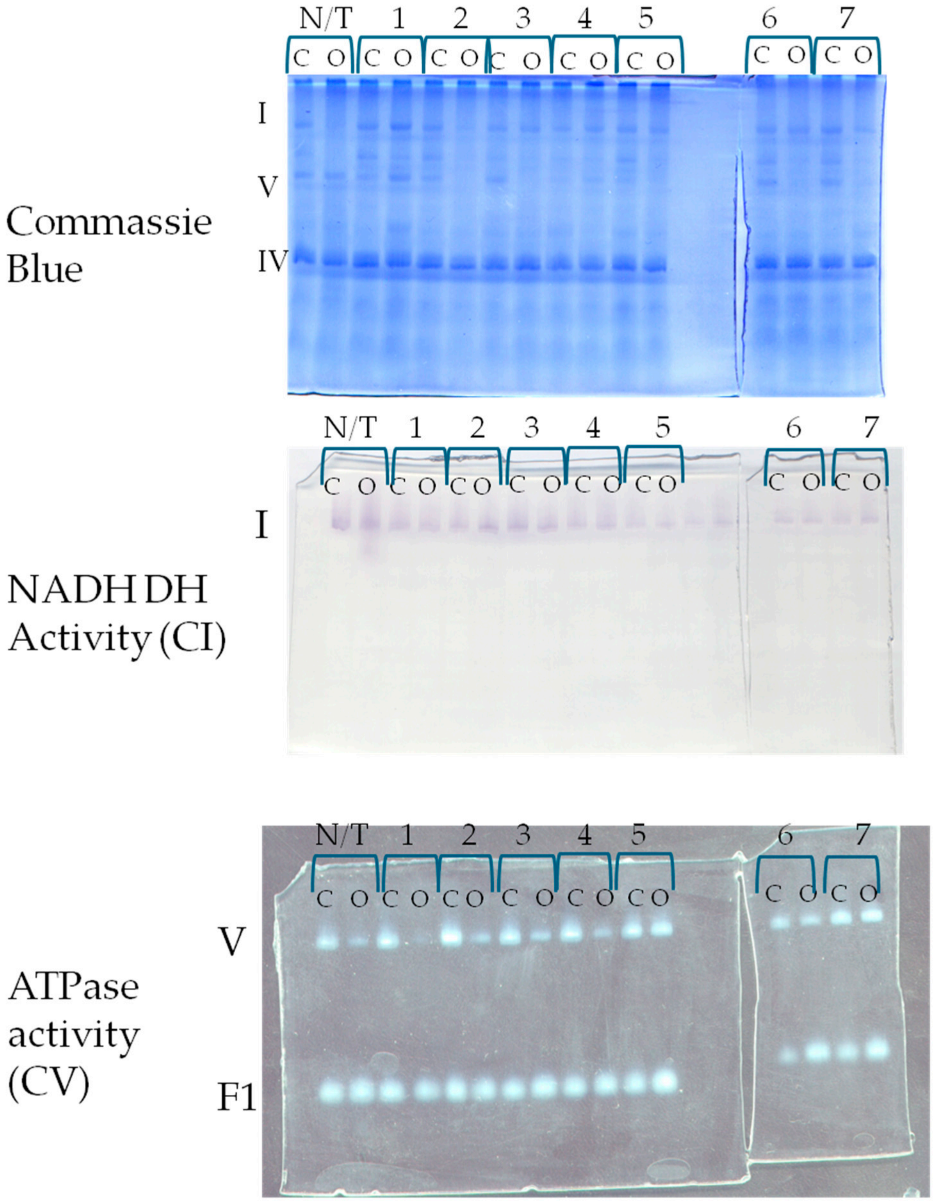

N/T: non-treated

1) 2-hydroxyestrone (2OHE1)
2) estrone-3-methyl-ether (3MOE1)
3) 4-methoxy-β-estradiol (4MEOE2)
4) 17-β-estradiol-3-methyl-ether (3MEOE2)
5) Estriol (E3)
6) 17β-estradiol (E2).
7) EDM mix

C: Ctrl
Oopho: Oophorectomized

Figure S2. Concentration and activity of mitochondrial redox complexes
